# Relative Leukocyte Telomere Length Is Associated with Multimorbidity Burden in Older Adults: Evidence for Sex-Specific Associations

**DOI:** 10.3390/ijms27104465

**Published:** 2026-05-16

**Authors:** Rossella La Grotta, Paolina Crocco, Aleksandra Leonova, Serena Dato, Giuseppe Passarino, Giuseppina Rose

**Affiliations:** Department of Biology, Ecology and Earth Sciences, University of Calabria, 87036 Rende, Italy; rossella.lagrotta@unical.it (R.L.G.); paolina.crocco@unical.it (P.C.); aleksandra.leonova@unical.it (A.L.); serena.dato@unical.it (S.D.); giuseppe.passarino@unical.it (G.P.)

**Keywords:** telomere length, multimorbidity, Cumulative Illness Rating Scale (CIRS), biological aging, sex differences, cellular senescence, geriatric assessment

## Abstract

Leukocyte telomere length (LTL) has been proposed as a molecular marker of biological aging reflecting cumulative cellular stress and replicative senescence. Multimorbidity represents a major challenge in aging populations and reflects the progressive accumulation of chronic diseases. However, the relationship between LTL and multimorbidity burden remains incompletely understood. We investigated the association between LTL and multimorbidity burden, assessed using Cumulative Illness Rating Scale (CIRS) indices, in a cohort of older nursing home residents. Sex-stratified analyses were performed to explore potential biological heterogeneity. In multivariate analyses, shorter LTL was significantly associated with higher multimorbidity burden among women, particularly when considering severity- and comorbidity-weighted CIRS indices [False discovery rate-adjusted q-values (qFDR < 0.01)], whereas no significant associations were observed in men. Adjustment for functional status partially attenuated but did not eliminate these associations. Organ-specific analyses indicated that these associations in women were primarily driven by cardiovascular, respiratory, gastrointestinal, and genitourinary domains, systems commonly characterized by chronic inflammatory and oxidative stress processes that may promote telomere attrition. Overall, these findings support a sex-specific relationship between telomere dynamics and clinically relevant multimorbidity patterns in very old adults. LTL may reflect biologically meaningful aspects of disease severity and systemic stress regulation rather than merely the accumulation of diagnoses.

## 1. Introduction

Multimorbidity, defined as the coexistence of two or more chronic diseases within the same individual, has emerged as a major challenge in the context of global population aging [1]. Its prevalence rises sharply with age, affecting the majority of adults over 65 years and up to 80–90% of those aged 80 years or older [2]. Multimorbidity contributes substantially to frailty, disability, polypharmacy and mortality, thereby placing an increasing burden on healthcare systems. Despite its clinical and public health relevance, the biological mechanisms underpinning multimorbidity remain only partially understood.

Several risk factors, such as age, obesity, depression, and low educational level, have been consistently associated with an increased risk of accumulating chronic conditions over time [3]. These determinants, however, do not fully explain the wide interindividual variability in disease burden observed among older adults, suggesting that intrinsic biological processes may also contribute to the development of multimorbidity.

Aging is characterized by a progressive accumulation of molecular and cellular damage leading to the decline of multiple organ systems. Processes such as oxidative stress, chronic low-grade inflammation, mitochondrial dysfunction, and impaired DNA repair are thought to play major roles in this systemic deterioration [4]. The interplay among these mechanisms may account for the parallel development of several chronic conditions [5], a phenomenon sometimes referred to as “systemic aging” [6].

Identifying biomarkers that reflect the cumulative biological burden underlying multimorbidity may help clarify the mechanistic links between aging processes and disease accumulation.

Telomeres, the repetitive DNA–protein structures that cap the ends of linear chromosomes, play a critical role in maintaining genomic stability [7]. In addition to their structural role, telomere dynamics are influenced by molecular processes such as oxidative stress, inflammation, and DNA damage responses, which are central features of biological aging [8,9]. With each cell division, telomeres progressively shorten, ultimately triggering cellular senescence or apoptosis. Thus, leukocyte telomere length (LTL) is considered a marker of biological aging [10] and has been associated with a variety of age-related conditions, including cardiovascular disease, diabetes, neurodegenerative disorders [11,12,13,14], as well as mortality [15,16]. These observations suggest that telomere shortening may represent a common biological substrate linking aging with the accumulation of chronic diseases.

However, most previous studies have focused on single diseases or specific organ systems, rather than considering the overall burden of disease across multiple systems. Only a limited number of investigations have addressed the relationship between telomere length and multimorbidity as an integrated measure of health status. In a large population-based cohort, Niedzwiedz et al. [17] found no overall association between salivary telomere length and multimorbidity; however, in men, longer telomeres were linked to a lower risk of multimorbidity, which included mental health disorders. Conversely, Bernabeu-Wittel et al. [18] observed shorter telomeres in multimorbid patients who also exhibited sarcopenia or frailty, pointing to a possible interaction between telomere attrition and advanced biological aging.

Together, these findings highlight the complexity of the relationship between telomere length and multimorbidity and the potential influence of sex and clinical context.

Yet, the clinical relevance of telomere shortening as an indicator of multimorbidity burden remains incompletely understood, particularly in the context of well-documented sex differences in telomere dynamics and trajectories of age-related disease burden [19,20,21,22].

In the present study, we investigated the relationship between leukocyte telomere length (LTL) and multimorbidity burden in an elderly population. Multimorbidity was quantified using the Cumulative Illness Rating Scale (CIRS), a well-validated multidimensional tool commonly employed within the comprehensive geriatric assessment, which captures both the number and severity of chronic conditions across multiple organ systems [23]. We further examined whether the association between LTL and multimorbidity differed by sex.

Understanding how telomere dynamics relate to clinically relevant patterns of multimorbidity may provide insight into the molecular mechanisms linking cellular senescence, biological aging, and the accumulation of chronic diseases.

## 2. Results

### 2.1. Descriptive Analysis

Leukocyte telomere length (LTL) was measured in 511 nursing home residents (mean age: 82.48 ± 7.6 years), of whom 67.71% were women. The main demographic, clinical, and biochemical characteristics of the whole sample and stratified by sex are reported in Table 1.

In the overall sample, LTL was inversely correlated with chronological age (sex-adjusted partial r = −0.140; *p* = 0.002). When analyses were stratified by sex, this association remained statistically significant in women but not in men.

**Figure 1:**
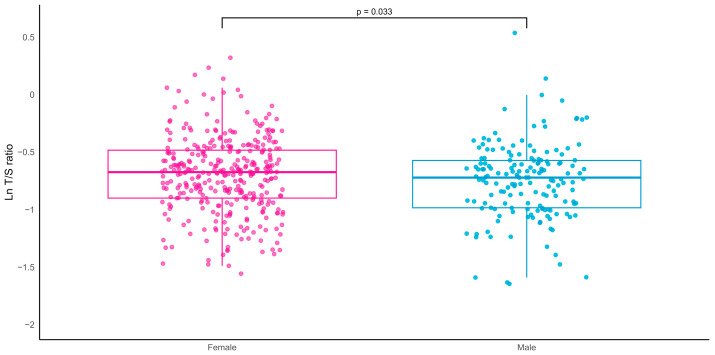
Mean values of peripheral blood leukocyte telomere length (LTL) measured as the logarithm of the number of copies of telomeric repeats (T) compared with a single-copy gene (S) (lnT/S ratio) in females and males. The *p*-value indicates the significance of the difference after adjusting for age.

No significant sex differences were observed in multimorbidity burden, as assessed by the CIRS total score (CIRS-TS), severity index (CIRS-SI), or comorbidity index (CIRS-CI). In contrast, significant differences between sexes were observed in selected anthropometric, metabolic, and functional parameters. Mean LTL was significantly shorter in men than in women (Figure 1; *p* = 0.033).

### 2.2. Association Between Telomere Length and Multimorbidity Burden (CIRS-14 Items)

Given the well-documented sex differences in telomere biology and aging trajectories, as well as the sex-specific patterns observed in LTL distribution and age association within our cohort, analyses were conducted both in the overall sample and stratified by sex.

In the overall sample, shorter LTL was significantly associated with higher multimorbidity burden, as assessed by the CIRS total score, severity index, and comorbidity index (Table 2). These associations were observed after adjustment for age and sex (Model 1) and remained significant after additional adjustment for BMI, albumin, and C-reactive protein (Model 2). However, sex-stratified analyses revealed a sex-specific pattern. In women, shorter LTL was consistently associated with higher values of all CIRS indices across both models. In contrast, no statistically significant associations were observed in men.

Among women, the strongest associations were observed for the CIRS severity index and comorbidity index, indicating that the strength of the association was greater for indices reflecting disease severity and clinical burden than for the total number of chronic conditions.

The robustness of the findings was further supported by false discovery rate (FDR) correction across the CIRS indices (qFDR ≤ 0.01).

Sensitivity analyses were additionally performed excluding the psychiatric/behavioral domain (CIRS-13), given the high prevalence of cognitive impairment and neuropsychiatric conditions in very old, institutionalized populations and their potential to disproportionately influence multimorbidity scoring. Results were highly comparable to those obtained with the CIRS-14, with similar magnitude and direction of the associations across all indices (Supplementary Table S1), indicating that the observed relationships were not driven by psychiatric morbidity.

### 2.3. Telomere Length and Individual CIRS Organ Systems in Women

To further explore which disease domains contributed to the observed associations, analyses were conducted at the level of individual CIRS organ systems among women. As reported in Table 3, shorter LTL was associated with higher severity scores in multiple systems, including cardiovascular, vascular, respiratory, gastrointestinal, hepatic, and genitourinary domains. No significant associations were observed for renal, endocrine–metabolic, or psychiatric/behavioral systems.

These findings indicate that the association between telomere length and multimorbidity in women was distributed across multiple somatic domains rather than being driven by a single organ system.

### 2.4. Secondary Analyses

#### 2.4.1. Severe Multimorbidity (CIRS-CI ≥ 2)

To further assess the clinical relevance of the observed associations, secondary analyses were conducted using a binary definition of severe multimorbidity, defined as the presence of at least two chronic conditions rated as severe (CIRS-CI ≥ 2). Logistic regression models showed that longer telomere length was associated with lower odds of severe multimorbidity in women (OR = 0.389, 95% CI = 0.187–0.773, *p* = 0.008), independent of age, BMI, albumin, and C-reactive protein. No association was observed in men.

Additional exploratory analyses comparing women at the extremes of the telomere length distribution showed that participants with shorter telomeres (<first quartile) had consistently higher multimorbidity burden across all CIRS indices compared with those with longer telomeres (>third quartile) (Supplementary Table S2). Logistic regression analyses further indicated that higher multimorbidity burden was associated with lower odds of belonging to the longer telomere group.

Taken together, these findings from both continuous and categorical analyses support an association between shorter telomeres and clinically relevant multimorbidity, particularly among women.

#### 2.4.2. Association with Functional and Cognitive Measures

Because multimorbidity is closely intertwined with functional and cognitive status in older adults, we further examined the relationships between telomere length, multimorbidity indices, and measures of function and cognition.

Multimorbidity indices were consistently and inversely associated with functional and cognitive measures (Supplementary Table S3). Higher CIRS scores were associated with lower activities of daily living (ADL) performance and lower mini-mental state examination (MMSE) scores, with similar patterns observed in both sexes. Higher multimorbidity burden was associated with reduced handgrip strength (HGS) only in women. As shown in Supplementary Table S4, LTL was positively associated with functional measures in women, particularly ADL (β = 0.144, *p* = 0.010) and HGS (β = 0.127, *p* = 0.037) in age-adjusted models. These associations were attenuated and no longer statistically significant after further adjustment for clinical covariates. No significant associations were observed in men or in the overall sample, and LTL was not associated with MMSE. When functional measures were included in multivariable models (Supplementary Table S5), the association between telomere length and multimorbidity indices was partially attenuated. Adjustment for ADL reduced the strength of the associations, whereas inclusion of HGS did not fully account for them, indicating that functional status explains part, but not all, of the observed relationship between telomere length and multimorbidity.

#### 2.4.3. Stratified Analyses by Age Tertiles

To explore potential age-related differences, analyses were stratified by age tertiles in women. Inverse associations between telomere length and the CIRS severity index were observed across tertiles, with slightly larger effect estimates in the oldest group. Associations with the comorbidity index reached statistical significance only in the highest tertile (β = −0.024, *p* = 0.016). Although exploratory, these findings are consistent with a potentially stronger link between telomere attrition and multimorbidity burden at more advanced ages.

## 3. Discussion

This study examined the relationship between leukocyte telomere length (LTL) and multimorbidity, measured by the Cumulative Illness Rating Scale (CIRS), in nursing home residents. We integrated LTL, a proxy for cellular aging and physiological stress [10,24], with multimorbidity, a hallmark of aging that reflects a progressive loss of physiological resilience [25]. Our goal was to determine if telomere shortening parallels systemic disease burden and functional vulnerability in the elderly. Ultimately, LTL may reflect life-course exposure to molecular stressors affecting telomere maintenance and cellular senescence.

A central finding of this study is a sex-specific association between LTL and multimorbidity. Specifically, shorter telomeres were associated with a higher disease burden in women, whereas no significant association was observed in men. This relationship was stronger when considering CIRS indices weighted for severity and comorbidity and was further confirmed by consistent results using binary definitions of severe multimorbidity.

Importantly, this pattern was not explained by differences in overall multimorbidity severity, as CIRS indices were comparable between women and men. Rather, these findings suggest that the biological significance and systemic context of telomere length may differ by sex.

Women in our cohort exhibited longer telomeres than men, and the inverse association between LTL and chronological age was observed only among women, supporting the presence of sex-related differences in telomere dynamics across aging. This interpretation aligns with large-scale studies reporting systematically longer telomeres in women and distinct patterns of telomere attrition across the life course [26,27], as well as broader evidence of sex differences in aging trajectories and morbidity accumulation [28]. Age-stratified analyses in women yielded comparable patterns across age tertiles.

Together, these findings suggest that sexual dimorphism in telomere biology and aging processes, rather than differences in disease burden per se, may underlie the observed sex-specific associations between telomere length and multimorbidity. Nevertheless, given the observational nature of the study and the absence of an independent validation cohort, these sex-specific findings should be considered exploratory and warrant confirmation in larger populations.

Potential biological mechanisms underlying this divergence include sex differences in inflammatory burden, innate and adaptive immune response, oxidative stress exposure, mitochondrial function, and long-term hormonal aging [29,30,31,32]. Estrogens, in particular, stimulate telomerase activity and exert antioxidant, anti-inflammatory, and immunoregulatory effects [33,34,35], promoting a closer link between telomere length and systemic health in women. These mechanisms may contribute to sex-specific differences in telomere maintenance with aging. As estrogen levels decline with advancing age, telomere length may become a more sensitive marker of accumulated physiological stress and reduced resilience in women. Conversely, the absence of significant associations in men may reflect a greater contribution of extrinsic risk factors (e.g., lifestyle and environmental exposures) or alternative, non-telomeric pathways of cellular senescence. A survival bias cannot be excluded, as men who reach very old age may represent a selected, resilient subgroup despite shorter baseline telomeres.

Within this sex-specific framework, the stronger association of LTL with the CIRS Severity and Comorbidity indices, compared with the total CIRS score, suggests that telomere shortening in women reflects clinical complexity and disease severity rather than merely the number of diagnoses. This observation is consistent with the concept of cumulative allostatic load, whereby severe and interacting chronic conditions impose sustained physiological strain through chronic inflammation, oxidative stress, and neuroendocrine dysregulation, processes central to inflammaging and telomere attrition [36,37]. Telomere shortening is a well-recognized trigger of replicative cellular senescence, thereby contributing to tissue dysfunction and systemic inflammatory amplification. Emerging evidence indicates that men and women differ not only in telomere biology but also in age-related patterns of allostatic load and stress-related physiological dysregulation, which may differentially shape the biological embedding of chronic disease across the life course [38]. In this context, our data suggest that the qualitative and severity-weighted burden of multimorbidity may be particularly relevant in shaping cellular aging trajectories in women.

Building on this interpretation, organ-specific analyses in women revealed that the association between telomere length and multimorbidity was not uniformly distributed across body systems, but was primarily driven by the cardiovascular, respiratory, gastrointestinal, and genitourinary domains.

This pattern suggests that telomere attrition, while reflecting systemic biological aging, may be more closely linked to specific pathogenic pathways characterized by sustained inflammatory and oxidative signaling rather than to overall disease accumulation per se. Cardiovascular and respiratory diseases are strongly characterized by chronic low-grade inflammation and oxidative stress [39,40,41], processes consistently implicated in telomere shortening [8,42,43]. Large population-based studies have reported shorter leukocyte telomeres in individuals with cardiovascular disease [13,44], and similar associations have been described in chronic pulmonary disorders [45,46]. Gastrointestinal and genitourinary conditions in older adults are likewise frequently associated with persistent inflammatory and immune dysregulation, biological environments that may contribute to telomere attrition [47,48]. Notably, several of these domains exhibit well-documented sexual dimorphism in prevalence, clinical expression, and inflammatory regulation [49,50,51,52], which may partly contextualize the stronger and more structured associations observed among women in our study. Overall, these findings are consistent with the possibility that telomere length is more strongly associated with multi-morbidity patterns characterized by inflammatory and oxidative stress–related mechanisms, rather than representing a nonspecific marker of disease burden.

The associations observed between multimorbidity indices and functional and cognitive measures provide additional insight into the clinical meaning of telomere shortening in late life. The consistent association between multimorbidity and reduced functional and cognitive performance (ADL, handgrip, and MMSE) underscores the close link between disease burden, functional vulnerability, and cognitive decline in older adults [53,54]. In parallel, LTL was positively associated with functional outcomes only among women, with no clear association with cognitive performance, suggesting that telomere length may capture sex-specific vulnerability to functional decline. This pattern may partly account for the heterogeneity reported in previous studies examining telomere length in relation to physical and cognitive outcomes [55,56,57].

The attenuation of the association between telomere length and multimorbidity after adjustment for ADL suggests that functional status shares part of the biological substrate linking telomere shortening and disease burden in late life, indicating partially overlapping mechanisms across cellular aging, multimorbidity, and functional decline. However, the persistence of significant associations after adjustment for handgrip strength indicates that telomere length is not merely a proxy of physical performance. Taken together, these findings indicate that telomere shortening is closely connected with both multimorbidity and functional vulnerability, while retaining associations not entirely explained by functional measures alone.

Interpretation of these findings in the context of the existing literature remains challenging because previous studies have adopted substantially different operational definitions of multimorbidity, ranging from simple disease counts to multidimensional severity-based instruments such as the CIRS. This methodological heterogeneity may partly contribute to the inconsistent associations reported between telomere length and multimorbidity across populations and clinical settings.

This study has several limitations. First, its cross-sectional design precludes conclusions regarding temporality or causality between telomere length and multimorbidity burden. Second, the relatively small number of men may have limited statistical power to detect sex-specific associations in this subgroup and reduced the ability to identify weaker associations in men. Therefore, the absence of statistically significant associations in men should not necessarily be interpreted as evidence of the absence of biological relationships. Third, although models were adjusted for major demographic, clinical, and inflammatory covariates, residual confounding from unmeasured or imprecisely measured variables cannot be excluded. In addition, telomere length was measured in DNA extracted from whole blood or buffy coat samples, precluding analyses of telomere dynamics in specific leukocyte subpopulations. Finally, the nursing home-based, very old population may limit generalizability to younger or community-dwelling cohorts.

## 4. Materials and Methods

### 4.1. Sample

The study sample included 511 nursing home residents (NHRs) aged 65 years and older (range 65–99 years, mean age 82.48 ± 7.62 years), of which 346 were females (mean age 82.58 ± 7.79) and 165 males (mean age 82.26 ± 7.26 years).

Following written informed consent from all participants or their legal representatives, recruited nursing home residents (NHRs) underwent a standardized, comprehensive geriatric assessment, which was performed systematically by trained personnel at the baseline visit. This evaluation included detailed clinical history, anthropometric data collection, and the administration of validated instruments to measure cognitive function, functional status, physical performance, nutritional condition, and depressive symptoms. In addition, common clinical hematological tests were performed.

The present study represents a cross-sectional observational analysis conducted within the framework of a broader research project on aging and longevity.

The study was approved by the Regional Ethics Committee of Catanzaro, Italy (code n. 25/2017) and was performed in accordance with the standards of ethics outlined in the Declaration of Helsinki.

### 4.2. Outcome

The main outcome of the present study was the cross-sectional association between telomere length and multimorbidity burden.

Multimorbidity was assessed using a modified version of the Cumulative Illness Rating Scale (CIRS) [23], a validated instrument that quantifies the overall burden of chronic medical illness across 14 organ systems (cardiac, vascular, respiratory, otorhinolaryngologic, upper gastrointestinal, lower gastrointestinal, hepatic/pancreatic, renal, genitourinary, musculoskeletal/dermatologic, neurological, endocrine/metabolic, breast, and psychiatric/behavioral).

Each system is rated from 0 (no problem) to 4 (severe impairment), yielding a total score ranging from 0 to 56, with higher values indicating greater morbidity burden.

From the CIRS, three standard indices were derived: the total score (CIRS-TS), corresponding to the sum of the 14 system scores; the severity index (CIRS-SI), calculated as the mean of the system scores; and the comorbidity index (CIRS-CI), defined as the number of organ systems with a severity rating ≥ 3. For descriptive purposes, the presence of at least two severe conditions (CIRS-CI ≥ 2) was also considered.

Secondary CIRS indices excluding the psychiatric/behavioral domain (CIRS-13) were computed to evaluate whether associations were driven by somatic disease burden.

### 4.3. Comprehensive Geriatric Assessment

Everyday functioning was assessed using the activities of daily living (ADL) scale [58]. The score was determined by counting the number of activities (bathing, dressing, toileting, eating, and transferring in and out of bed) in which the participant was independent at the time of the visit. Each activity was scored as 0 if the participant was unable to perform the task and 1 if they were able to perform such an activity. ADL scores therefore ranged from 0 (unable to perform any activity) to 5 (able to perform all activities).

Muscle strength was evaluated through handgrip strength (HGS), measured with a handheld dynamometer (SMEDLEY’s dynamometer TTM) while participants were seated with the tested arm held close to the body. The assessment was performed three times using the dominant hand, and the highest value obtained was used for analysis.

The mini-mental state examination (MMSE) tool was used to assess cognitive function, evaluating orientation, episodic memory, attention, language, and construction functions [59]. The MMSE scores (from 0 to 30) were age- and education-adjusted. Patients scoring less than 24 were considered cognitively impaired.

### 4.4. Measurements of Leukocyte Telomere Length (LTL)

Genomic DNA was isolated from whole blood or buffy coat using the salting-out procedure described by Miller and colleagues [60]. Leukocyte telomere length (LTL) was subsequently determined by quantitative real-time PCR (qPCR) according to the method originally described by Cawthon, with modifications by Testa and colleagues [61,62]. This approach evaluates relative telomere length by calculating the T/S ratio, which represents the number of telomeric repeat copies (T) relative to a single-copy reference gene (S).

The single-copy gene used as an internal control was 36B4, encoding the acidic ribosomal phosphoprotein P0. For each DNA sample, telomeric repeats (T) and the 36B4 gene (S) were amplified in triplicate in separate reactions performed on the same plate in order to minimize inter-assay variability. The primer sequences used (5′→3′) were: tel1, GGTTTTTGAGGGTGAGGGTGAGGGTGAGGGTGAGGGT; tel2, TCCCGACTATCCCTATCCCTATCCCTATCCCTATCCCTA; 36B4u, CAGCAAGTGGGAAGGTGTAATCC; and 36B4d, CCCATTCTATCATCAACGGGTACAA.

Each reaction was carried out in a final volume of 20 µL containing 15 µL of master mix (including PCR reagents, SYBR Green dye, and specific primers for either telomeres or 36B4) and 5 µL of genomic DNA at a concentration of 3 ng/µL (15 ng total DNA per reaction). A calibrator DNA sample (Roche, Milan, Italy; 5 µL at 3 ng/µL) was included in every plate to allow comparison between runs. In addition, two standard curves (one for telomeres and one for 36B4) were generated on each plate using a reference DNA sample (Roche, Milan, Italy) serially diluted by 1.68-fold to obtain six DNA concentrations ranging from 30 to 2 ng per 5 µL.

Amplifications were performed in 96-well plates using a QuantStudio 3 Real-Time PCR System (Applied Biosystems, Thermo Fisher Scientific, Waltham, MA, USA) with the following thermal cycling profile: an initial denaturation at 95 °C for 10 s followed by 30 cycles of 95 °C for 5 s, 57 °C for 15 s, and 72 °C for 20 s. Relative telomere length was expressed as the T/S ratio normalized to the calibrator sample.

To assess assay reproducibility, more than 20% of samples were randomly replicated on independent plates. The standard curves yielded average slopes of −3.10 for telomeres and −3.21 for 36B4, with correlation coefficients () consistently ranging from 0.98 to 0.99. Mean amplification efficiencies were within the acceptable range for both telomere and 36B4 assays (approximately 106% and 105%, respectively). All unknown samples yielded Cq values within the linear dynamic range of the corresponding standard curves. Representative calibration curves for telomere and 36B4 amplification are provided in Supplementary Figure S1. The inter-assay coefficient of variation was below 8%.

### 4.5. Statistical Analysis

Descriptive statistics summarize demographic and clinical characteristics. Normality was assessed using the Shapiro–Wilk test. Continuous variables are reported as mean ± SD and categorical variables as frequencies and percentages. Group comparisons were performed using Student’s *t* test or Mann–Whitney U test for continuous variables and χ^2^ test for categorical variables.

Leukocyte telomere length (LTL) was log-transformed and analyzed as a continuous variable. Associations between LTL and multimorbidity burden, assessed using CIRS-14 indices (total score, severity index, and comorbidity index), were examined using linear regression models in the whole sample and stratified by sex. Models were adjusted for age and sex (or age only in sex-stratified analyses), with additional adjustment for BMI, serum albumin, and C-reactive protein in fully adjusted models. Model assumptions were evaluated by inspection of residuals and assessment of multicollinearity.

Organ-specific CIRS domains were analyzed using similar regression models. Severe multimorbidity (CIRS-CI ≥ 2) was evaluated using logistic regression models adjusted for the same covariates. Additional exploratory analyses were performed in women comparing participants at the extremes of the telomere length distribution (<1st quartile vs. >3rd quartile). Logistic regression models were used with telomere length group as the dependent variable and multimorbidity indices as predictors, adjusting for age, BMI, albumin, and C-reactive protein.

Associations with functional and cognitive measures (ADL, HGS, and MMSE) were assessed using linear regression models.

Sensitivity analyses were performed using the CIRS-13 score. Exploratory age-stratified analyses were conducted in women.

Analyses were conducted using SPSS v30.0 (IBM Corp., NY, USA). A two-sided *p*-value < 0.05 was considered statistically significant. False discovery rate (FDR) correction was applied across the three primary CIRS indices within each analytical set.

## 5. Conclusions

Our findings indicate that leukocyte telomere length is associated with the burden and severity of multimorbidity in very old adults, particularly among women. Telomere shortening may capture biologically relevant aspects of disease complexity and functional vulnerability rather than merely reflecting the accumulation of diagnoses. These results support the interpretation of telomere length as a potential integrative marker linking cellular senescence, pathogenic mechanisms, and clinical multimorbidity in late life. The observed sex-specific patterns further suggest that regulatory mechanisms governing telomere maintenance and stress responsiveness may differentially shape aging trajectories in women and men. Future mechanistic and longitudinal studies will be essential to clarify whether telomere shortening acts solely as a biomarker or also contributes to the biological pathways driving multimorbidity progression in late life, as well as to explore its future therapeutic potential. Clarifying these mechanisms may help to identify molecular pathways linking telomere dysfunction with systemic aging processes and could contribute to the development of future biomarkers of biological aging and disease vulnerability.

## Figures and Tables

**Table 1:** Baseline demographic, clinical, and functional characteristics of the study population overall and stratified by sex.

Parameters	Whole Sample (*n* = 511)	Females (*n* = 346)	Males (*n* = 165)	*p*-Values
Age, years	82.48 (7.62)	82.58 (7.79)	82.26 (7.26)	0.70
Multimorbidity				
CIRS-TS	16.25 (12.77)	16.00 (13.19)	16.76 (11.85)	0.128
CIRS-SI	1.88 (0.61)	1.89 (0.63)	1.87 (0.55)	0.834
CIRS-CI	2.26 (3.18)	2.27 (3.30)	2.25 (2.93)	0.417
Anthropometric				
BMI, kg/m^2^	25.88 (4.99)	25.57 (5.41)	26.52 (3.93)	<0.001
Height, m	1.55 (0.09)	1.52 (0.08)	1.61 (0.08)	<0.001
WHR	0.93 (0.071)	0.91 (0.072)	0.96 (0.058)	<0.001
Biochemical				
FPG, mg/dL	102.36 (40.90)	102.23 (41.59)	102.63 (39.71)	0.556
HbA1c, %	6.03 (1.42)	6.03 (1.44)	6.06 (1.40)	0.588
TC, mg/dL	160.08 (39.85)	165.33 (39.56)	149.53 (38.46)	<0.001
HDL-C, mg/dL	49.94 (16.42)	51.81 (17.78)	45.95 (12.17)	<0.001
LDL-C, mg/dL	89.74 (32.12)	92.90 (31.45)	83.02 (32.64)	0.005
TG, mg/dL	122.94 (67.01)	122.94 (66.35)	122.93 (68.57)	0.945
Albumin, g/dL	52.90 (8.95)	53.49 (6.16)	51.68 (12.94)	0.689
Total protein, g/dL	6.53(0.62)	6.53 (0.63)	6.54 (0.63)	0.698
CRP, mg/dL	10.35 (20.97)	10.26 (20.82)	12.54 (21.35)	0.511
Creatinine, mg/dL	1.10 (0.47)	1.05 (0.45)	1.23 (0.49)	<0.001
Uric acid (mg/dL)	4.84 (1.48)	4.71 (1.48)	5.11 (1.43)	0.001
Azotemia (mg/dL)	50.62 (26.05)	50.48 (26.73)	50.89 (27.74)	0.281
Hematological				
RBC, ×10^12^/L	5.02 (16.52)	5.35 (20.03)	4.32 (0.72)	0.069
WBC, ×10^9^/L	6.83 (2.54)	6.77 (2.61)	6.98 (2.36)	0.157
Neutrophils, %	58.29 (15.13)	57.98 (10.46)	58.91 (12.42)	0.131
Lymphocytes, %	25.77(9.66)	26.95 (9.91)	23.39 (8.71)	<0.001
Monocytes, %	10.57 (5.61)	10.00 (4.99)	11.74 (6.57)	0.005
Basophils, %	0.82 (0.54)	0.84 (0.54)	0.78 (0.54)	0.202
Eosinophils, %	2.69 (2.29)	2.69 (2.40)	2.68 (2.06)	0.810
Platelets, ×10^9^/L	241.01 (95.49)	254.34 (100.23)	214.37 (79.02)	<0.001
Functional				
ADL	2.27 (1.93)	2.07 (1.90)	2.70 (1.93)	<0.001
HGS	15.20 (8.15)	12.67 (6.39)	19.90 (8.95)	<0.001
MMSE	18.08 (6.29)	17.30 (6.28)	19.69 (6.03)	<0.001

Continuous variables are expressed as mean and standard deviation (SD), while categorical variables are expressed as percentages (%). *p*-values are derived from the *t*-test or Mann–Whitney test, depending on continuous data distribution, and from the chi-squared test of association for categorical variables. *Abbreviations*: CIRS-TS, Cumulative Illness Rating Scale (CIRS)—Total Score; CIRS-SI, Cumulative Illness. Rating Scale (CIRS)—Severity Index; CIRS-CI, Cumulative Illness Rating Scale (CIRS)—Comorbidity Index. BMI, body mass index; WHR, waist-to-hip ratio; FPG, fasting plasma glucose; HbA1c, glycosylated hemoglobin; TC, total cholesterol; HDL-C, high-density lipoprotein cholesterol; LDL-C, low-density lipoprotein cholesterol; TGs, triglycerides; CRP, C-reactive protein; RBC, red blood cell; WBC, white blood cell; ADLs, activities of daily living; HGS, handgrip strength; MMSE, mini-mental state examination.

**Table 2:** Association between leukocyte telomere length and multimorbidity indices (CIRS-14).

	Model	Whole Sample β (SE)	*p*-Value	qFDR	Female β (SE)	*p*-Value	qFDR	Male β (SE)	*p*-Value
CIRS-TS	Model 1	−0.003 (0.001)	0.007	0.011	−0.004 (0.001)	0.004	0.009	−0.002 (0.003)	ns
	Model 2	−0.003 (0.001)	0.005	0.009	−0.004 (0.001)	0.007	0.011	−0.002 (0.003)	ns
CIRS-SI	Model 1	−0.078 (0.027)	0.004	0.009	−0.114 (0.029)	<0.001	0.001	0.018 (0.057)	ns
	Model 2	−0.079 (0.027)	0.004	0.009	−0.111 (0.029)	<0.001	0.001	0.029 (0.058)	ns
CIRS-CI	Model 1	−0.015 (0.005)	0.005	0.009	−0.019 (0.006)	0.001	0.006	−0.003 (0.011)	ns
	Model 2	−0.015 (0.005)	0.004	0.009	−0.018 (0.006)	0.002	0.007	−0.001 (0.011)	ns

CIRS-TS, Cumulative Illness Rating Scale (CIRS)—Total Score; CIRS-SI, Cumulative Illness Rating Scale (CIRS)—Severity Index; CIRS-CI, Cumulative Illness Rating Scale (CIRS)—Comorbidity Index. Model 1: adjusted for age and sex in the whole sample; adjusted for age in sex-stratified analyses. Model 2: adjusted for age, sex, body mass index (BMI), serum albumin, and C-reactive protein (CRP) in the whole sample; adjusted for age, BMI, serum albumin, and CRP in sex-stratified analyses. β (SE) indicates the unstandardized regression coefficient and standard error. qFDR: false discovery rate; adjusted *p*-values: not statistically significant (*p* ≥ 0.05).

**Table 3:** Association between leukocyte telomere length (LTL) and Cumulative Illness Rating Scale (CIRS) organ-specific domains in women.

CIRS—Items	Beta (SE)	*p*-Value
Cardiac (heart only)	−0.045 (0.014)	0.001
Hypertension (rating is based on severity; organ damage is rated separately)	−0.030 (0.016)	0.059
Vascular (blood, blood vessels and cells, bone marrow, spleen, and lymphatics)	−0.045 (0.014)	0.001
Respiratory (lungs, bronchi, and trachea below the larynx)	−0.032 (0.016)	0.048
EENT (eye, ear, nose, throat, and larynx)	−0.037 (0.017)	0.028
Upper GI (esophagus, stomach, duodenum, and pancreas)	−0.063 (0.018)	<0.001
Lower GI (intestines and hernias)	−0.041 (0.018)	0.025
Hepatic (liver and biliary tree)	−0.045 (0.019)	0.016
Renal (kidneys only)	−0.016 (0.017)	0.338
Other GU (uterus, bladder, urethra, prostate, and genitals)	−0.042 (0.018)	0.021
Musculoskeletal–integumentary (muscle, bone, and skin)	−0.029 (0.015)	0.062
Neurological (brain, spinal cord, and nerves; does not include dementia)	−0.028 (0.014)	0.050
Endocrine–metabolic (diabetes, thyroid; breast; systemic infections; toxicity)	−0.012 (0.016)	0.458
Psychiatric/behavioral (dementia, depression, anxiety, agitation/delirium, psychosis)	−0.016 (0.014)	0.236

Beta coefficients are presented with standard errors (SEs). *p*-values refer to regression coefficients derived from linear models adjusted for age, body mass index (BMI), serum albumin, and C-reactive protein (CRP).

## Data Availability

The raw datasets are not included in the Supplementary Material and are available only upon reasonable request.

## Supplementary Materials

The following supporting information can be downloaded at: https://www.mdpi.com/article/10.3390/ijms27104465/s1.
