# Supplementary material for: Relative Leukocyte Telomere Length Is Associated with Multimorbidity Burden in Older Adults: Evidence for Sex-Specific Associations
Source: Int J Mol Sci. 2026 May 16;27(10):4465. doi: 10.3390/ijms27104465 (PMC13207454; doi:10.3390/ijms27104465)
Supplement: Supplementary file 1 [file ijms-27-04465-s001.zip › Supplementary Table S1.pdf]

Supplementary Table S1. Association between leukocyte telomere length and multimorbidity indices derived from the Cumulative Illness Rating Scale excluding the psychiatric domain (CIRS-13).

	Model	Whole sample β (SE)	p-value	Female β (SE)	p-value	Male β (SE)	p-value
CIRS-TS	Model 1	-0.004 (0.001)	0.005	-0.003 (0.002)	0.003	-0.002 (0.003)	ns
	Model 2	-0.004 (0.001)	0.004	-0.004 (0.002)	0.006	-0.002 (0.003)	ns
CIRS-SI	Model 1	-0.068 (0.028)	0.008	-0.094 (0.028)	<0.001	0.005 (0.054)	ns
	Model 2	-0.068 (0.028)	0.008	-0.088 (0.028)	0.002	0.011 (0.055)	ns
CIRS-CI	Model 1	-0.017 (0.006)	0.002	-0.021 (0.006)	<0.001	-0.005 (0.012)	ns
	Model 2	-0.018 (0.006)	0.002	-0.020 (0.006)	0.001	-0.005 (0.012)	ns

CIRS-TS, Cumulative Illness Rating Scale (CIRS)-Total Score; CIRS-SI, Cumulative Illness Rating Scale (CIRS)-Severity Index; CIRS-CI, Cumulative Illness Rating Scale (CIRS)-Comorbidity Index.
Model 1: adjusted for age and sex in the whole sample; adjusted for age in sex-stratified analyses.
Model 2: adjusted for age, sex, body mass index (BMI), serum albumin, and C-reactive protein (CRP) in the whole sample; adjusted for age, BMI, serum albumin, and CRP in sex-stratified analyses.
β (SE) indicates the unstandardized regression coefficient and standard error.
ns: not statistically significant ($p \geq 0.05$).
