# Supplementary material for: Relative Leukocyte Telomere Length Is Associated with Multimorbidity Burden in Older Adults: Evidence for Sex-Specific Associations
Source: Int J Mol Sci. 2026 May 16;27(10):4465. doi: 10.3390/ijms27104465 (PMC13207454; doi:10.3390/ijms27104465)
Supplement: Supplementary file 1 [file ijms-27-04465-s001.zip › Supplementary Table S2.pdf]

Supplementary Table S2. Multimorbidity burden according to extremes of leukocyte telomere length distribution in women.

CIRS index	Shorter LTL (<Q1) Mean (SD)	Longer LTL (>Q3) Mean (SD)	OR for belonging to longer LTL group (95% CI)*	p-value
CIRS-TS	19.43 (14.88)	13.61 (11.10)	0.97 (0.94-0.99)	0.027
CIRS-SI	2.03 (0.69)	1.76 (0.54)	0.59 (0.31-0.92)	0.024
CIRS-CI	3.22 (3.92)	1.54 (2.51)	0.87 (0.78-0.96)	0.010

CIRS-TS, Cumulative Illness Rating Scale (CIRS)-Total Score; CIRS-SI, Cumulative Illness Rating Scale (CIRS)- Severity Index; CIRS-CI, Cumulative Illness Rating Scale (CIRS)-Comorbidity Index.
*Odds ratios derived from logistic regression models adjusted for age, BMI, albumin, and C-reactive protein. The dependent variable was telomere length group (shorter vs. longer telomeres).
