# Supplementary material for: Relative Leukocyte Telomere Length Is Associated with Multimorbidity Burden in Older Adults: Evidence for Sex-Specific Associations
Source: Int J Mol Sci. 2026 May 16;27(10):4465. doi: 10.3390/ijms27104465 (PMC13207454; doi:10.3390/ijms27104465)
Supplement: Supplementary file 1 [file ijms-27-04465-s001.zip › Supplementary Table S3.pdf]

Supplementary Table S3. Association between multimorbidity indices and functional and cognitive measures.

	CIRS index	Whole sample β (p)	Female β (p)	Male β (p)
**ADL**	CIRS-TS	-0.358 (<0.001)	-0.382 (<0.001)	-0.319 (<0.001)
	CIRS-SI	-0.354 (<0.001)	-0.353 (<0.001)	-0.372 (<0.001)
	CIRS-CI	-0.359 (<0.001)	-0.365 (<0.001)	-0.363 (<0.001)
**HGS**	CIRS-TS	-0.152 (<0.001)	-0.204 (<0.001)	-0.124 (0.118)
	CIRS-SI	-0.131 (0.002)	-0.202 (<0.001)	-0.061(0.439)
	CIRS-CI	-0.150 (<0.001)	-0.215 (<0.001)	-0.105 (0.185)
**MMSE**	CIRS-TS	-0.227 (<0.001)	-0.240 (<0.001)	-0.206 (0.010)
	CIRS-SI	-0.230 (<0.001)	-0.258 (<0.001)	-0.173 (0.031)
	CIRS-CI	-0.211 (<0.001)	-0.226 (<0.001)	-0.188 (0.020)

Values are standardized β coefficients from linear regression models adjusted for age, sex, body mass index (BMI), serum albumin, and C-reactive protein (CRP) in the whole sample; adjusted for age, BMI, serum albumin, and CRP in sex-stratified analyses.
Abbreviations: ADL, activities of daily living; HGS, handgrip strength; MMSE, Mini-Mental State Examination.
