# Supplementary material for: Relative Leukocyte Telomere Length Is Associated with Multimorbidity Burden in Older Adults: Evidence for Sex-Specific Associations
Source: Int J Mol Sci. 2026 May 16;27(10):4465. doi: 10.3390/ijms27104465 (PMC13207454; doi:10.3390/ijms27104465)
Supplement: Supplementary file 1 [file ijms-27-04465-s001.zip › Supplementary Table S4.pdf]

Supplementary Table S4. Association between leukocyte telomere length and functional and cognitive measures in the whole sample and stratified by sex.

	Whole sample β (p)	Female β (p)	Male β (p)
**ADL**	0.071 (0.135)	0.144 (0.010)	-0.062 (0.457)
**HGS**	0.035 (0.526)	0.127 (0.037)	-0.067 (0.447)
**MMSE**	-0.011 (0.847)	0.063 (0.334)	-0.146 (0.144)

Values are standardized β coefficients from linear regression models adjusted for age and sex in the whole sample and for age in sex-stratified analyses.
Abbreviations: ADL, activities of daily living; HGS, handgrip strength; MMSE, Mini-Mental State Examination.
