# Supplementary material for: Relative Leukocyte Telomere Length Is Associated with Multimorbidity Burden in Older Adults: Evidence for Sex-Specific Associations
Source: Int J Mol Sci. 2026 May 16;27(10):4465. doi: 10.3390/ijms27104465 (PMC13207454; doi:10.3390/ijms27104465)
Supplement: Supplementary file 1 [file ijms-27-04465-s001.zip › Supplementary Table S5.pdf]

Supplementary Table S5. Associations between leukocyte telomere length, multimorbidity indices, and functional and cognitive measures (ADL, MMSE, HGS) in women.

	Model 1 + ADL β (p-value)	Model 2 + ADL β (p-value)	Model 1 + HGS β (p-value)	Model 2 + HGS β (p-value)	Model 1 + ADL + HGS β (p-value)	Model 2+ ADL + HGS β (p-value)
CIRS-TS	-0.102 (0.044)	-0.099 (0.053)	-0.200 (0.001)	-0.204 (0.001)	-0.154 (0.008)	-0.161 (0.007)
CIRS-SI	-0.159 (0.002)	-0.163 (0.002)	-0.246 (<0.001)	-0.256 (<0.001)	-0.205 (<0.001)	-0.215 (<0.001)
CIRS-CI	-0.126 (0.014)	-0.128 (0.013)	-0.217 (<0.001)	-0.226 (<0.001)	-0.173 (0.003)	-0.184 (0.002)

Values are standardized regression coefficients (β) with corresponding p-values derived from linear regression models.
Model 1: adjusted for age
Model 2: adjusted for age, BMI, albumin, and C-reactive protein.
Abbreviations: ADL, activities of daily living; HGS, handgrip strength; MMSE, Mini-Mental State Examination; CIRS-TS, Cumulative Illness Rating Scale (CIRS)-Total Score; CIRS-SI, Cumulative Illness Rating Scale (CIRS)-Severity Index; CIRS-CI, Cumulative Illness Rating Scale (CIRS)-Comorbidity Index.
