# Supplementary figures and images for: Relative Leukocyte Telomere Length Is Associated with Multimorbidity Burden in Older Adults: Evidence for Sex-Specific Associations
Source: Int J Mol Sci. 2026 May 16;27(10):4465. doi: 10.3390/ijms27104465 (PMC13207454; doi:10.3390/ijms27104465)

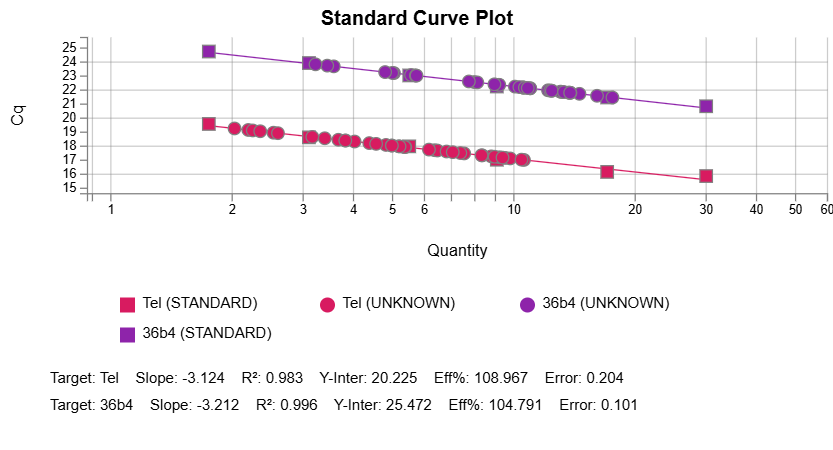

Supplement: Supplementary file 1 [file ijms-27-04465-s001.zip › Supplementary Figure S1.png]
